# Assembly and Succession of Iron Oxide Microbial Mat Communities in Acidic Geothermal Springs

**DOI:** 10.3389/fmicb.2016.00025

**Published:** 2016-02-15

**Authors:** Jacob P. Beam, Hans C. Bernstein, Zackary J. Jay, Mark A. Kozubal, Ryan deM. Jennings, Susannah G. Tringe, William P. Inskeep

**Affiliations:** ^1^Department of Land Resources and Environmental Sciences, Thermal Biology Institute, Montana State UniversityBozeman, MT, USA; ^2^Department of Chemical and Biological Engineering, Center for Biofilm Engineering, Montana State UniversityBozeman, MT, USA; ^3^Biodetection Science and Biological Science Division, Pacific Northwest National LaboratoryRichland, WA, USA; ^4^United States Department of Energy Joint Genome InstituteWalnut Creek, CA, USA

**Keywords:** *Hydrogenobaculum*, *Metallosphaera*, lithoautotroph, organoheterotroph, archaea, biomineralization, oxygen

## Abstract

Biomineralized ferric oxide microbial mats are ubiquitous features on Earth, are common in hot springs of Yellowstone National Park (YNP, WY, USA), and form due to direct interaction between microbial and physicochemical processes. The overall goal of this study was to determine the contribution of different community members to the assembly and succession of acidic high-temperature Fe(III)-oxide mat ecosystems. Spatial and temporal changes in Fe(III)-oxide accretion and the abundance of relevant community members were monitored over 70 days using sterile glass microscope slides incubated in the outflow channels of two acidic geothermal springs (pH = 3–3.5; temperature = 68–75°C) in YNP. *Hydrogenobaculum* spp. were the most abundant taxon identified during early successional stages (4–40 days), and have been shown to oxidize arsenite, sulfide, and hydrogen coupled to oxygen reduction. Iron-oxidizing populations of *Metallosphaera yellowstonensis* were detected within 4 days, and reached steady-state levels within 14–30 days, corresponding to visible Fe(III)-oxide accretion. Heterotrophic archaea colonized near 30 days, and emerged as the dominant functional guild after 70 days and in mature Fe(III)-oxide mats (1–2 cm thick). First-order rate constants of Fe(III)-oxide accretion ranged from 0.046 to 0.05 day^−1^, and *in situ* microelectrode measurements showed that the oxidation of Fe(II) is limited by the diffusion of O_2_ into the Fe(III)-oxide mat. The formation of microterracettes also implicated O_2_ as a major variable controlling microbial growth and subsequent mat morphology. The assembly and succession of Fe(III)-oxide mat communities follows a repeatable pattern of colonization by lithoautotrophic organisms, and the subsequent growth of diverse organoheterotrophs. The unique geochemical signatures and micromorphology of extant biomineralized Fe(III)-oxide mats are also useful for understanding other Fe(II)-oxidizing systems.

## Introduction

Microbial mat communities are ubiquitous geobiological features (e.g., stromatolites; Riding, 1999) in contemporary and past environments on Earth, are often stratified due to gradients in key geochemical constituents (e.g., oxygen; de Beer et al., 1994; Bernstein et al., 2013), and often leave biological signatures (biomarkers) preserved in the rock record (e.g., lipids; Peters and Moldowan, 1993). Biogeochemical stratification of microbial mats may produce distinct morphological features that can be preserved (e.g., iron formations and marine carbonates), and provide insights into past geochemical and hydrodynamic conditions (e.g., Kappler and Straub, 2005; Fouke, 2011). Microbial mat communities often produce extracellular polymeric substances (EPS), which may serve as sites for mineral nucleation and growth (e.g., Mann, 1988; Kandianis et al., 2008). Understanding mechanisms of assembly and succession in modern-day mat communities provides clues regarding past environmental conditions and biogeophysical controls that lead to the biomineralization of specific solid phases (Reid et al., 2000; Fouke, 2011). Moreover, modern microbial mat ecosystems can be observed in real-time and monitored over spatial and temporal scales to elucidate mechanisms of formation under various geochemical and hydrologic conditions.

Iron is the fourth most abundant element in the Earth's crust and is an essential cofactor in numerous proteins across all domains of life (e.g., iron-sulfur proteins). Microorganisms may also gain energy via the oxidation of Fe(II) to Fe(III) under aerobic or anaerobic conditions, which usually results in the precipitation of insoluble solid-phase Fe(III)-oxides (Konhauser, 1998, 2006; Kappler and Straub, 2005; Ehrlich and Newman, 2009; Emerson, 2012). The reduction of Fe(III)-oxides to Fe(II) can be coupled with the oxidation of inorganic (e.g., hydrogen) or organic (e.g., acetate) compounds by other microorganisms, completing the Fe cycle. Uncatalyzed abiotic rates of Fe(II)-oxidation are extremely slow at pH values less than 4 (Singer and Stumm, 1970; Kappler and Straub, 2005); consequently, the deposition of Fe(III)-oxides under acidic conditions can often be attributed to the activity of Fe(II)-oxidizing microbial populations. Low pH (<4) acid-mine drainage (Denef et al., 2010) and acidic geothermal springs in YNP (Langner et al., 2001; Kozubal et al., 2012) exhibit significant amounts of Fe(III)-oxide biomineralization.

High-temperature (65–80°C), acidic (pH = 2–3.5) Fe(III)-oxide microbial mats in Yellowstone National Park (YNP) represent hydrodynamically-controlled model systems for studying microbial interactions and biogeochemical processes *in situ* (Kozubal et al., 2008, 2012; Inskeep et al., 2010, 2013). *Metallosphaera yellowstonensis* (order Sulfolobales, Crenarchaeota) is the primary chemolithoautotroph responsible for Fe(II)-oxidation in these systems, which results in the precipitation of copious amounts of Fe(III)-oxides and/or jarosite, depending on spring geochemistry (Kozubal et al., 2008, 2012). Protein-coding genes responsible for Fe(II)-oxidation in *M. yellowstonensis* (e.g., *foxC*) have been elucidated, and are highly expressed in Fe(III)-oxide microbial mats of YNP (Kozubal et al., 2011). *Hydrogenobaculum* spp. (order Aquificales) are versatile chemolithoautotrophs that inhabit sulfur and iron-dominated acidic hot springs, and have been shown to utilize several different electron donors (e.g., As(III), H_2_, and H_2_S) coupled with the reduction of O_2_ to drive the fixation of CO_2_ via the reductive TCA cycle (Donahoe-Christiansen et al., 2004; D'Imperio et al., 2007; Hamamura et al., 2009; Romano et al., 2013; Takacs-Vesbach et al., 2013). *Hydrogenobaculum* spp. and *Metallosphaera yellowstonensis* are both implicated as early colonizing populations (Macur et al., 2004) and carbon dioxide (CO_2_) fixing organisms (Jennings et al., 2014) in high-temperature Fe(III)-oxide mats of YNP; however, the role of these lithoautotrophs in early stages of Fe(III)-oxide mat formation has not been elucidated.

Prior work using stable carbon isotopes (^13^C) has shown that CO_2_-derived microbial C contributes a minimum of 40% of the total biomass C in mature Fe(III)-oxide mats (Jennings et al., 2014), which provides a significant organic C source for organoheterotrophic thermoacidophiles. Furthermore, *Hydrogenobaculum* spp. have also been shown to uptake radiolabeled bicarbonate (^14^CO_2_) in high sulfide zones of the same acidic geothermal springs (Boyd et al., 2009). Thus, the growth and assembly of early-colonizing lithoautotrophic populations (i.e., *Hydrogenobaculum* spp. and *Metallosphaera yellowstonensis*) will likely influence the subsequent succession and activity of organoheterotrophic organisms. Consequently, the primary objectives of the current study were to (i) determine the spatiotemporal dynamics of key community members involved in Fe(III)-oxide mat assembly in acidic geothermal springs of Norris Geyser Basin (YNP), (ii) quantify the amount of Fe(III)-oxides accreted and oxygen consumed *in situ* as a function of time, (iii) monitor temporal changes in community composition using 16S rRNA gene sequencing, and (iv) integrate laboratory measurements and field observations across different scales to develop a conceptual model of Fe(III)-oxide assembly and succession. A combination of geochemical, microscopic, and molecular methods were employed to reveal that *Hydrogenobaculum* spp. exhibit rapid growth rates *in situ* and are the first colonizers in high-temperature acidic Fe mats, followed by the accretion of Fe(III)-oxides due to the rise of Fe(II)-oxidizing populations of *Metallosphaera yellowstonensis*. Other heterotrophic archaea, which include several novel groups and additional Crenarchaeota (e.g., Sulfolobales, Desulfurococcales) colonize at later stages of mat development and likely utilize organic C produced by lithoautotrophs. Distinct stages of mat development were associated with specific micro-morphological features that provide a basis for understanding the assembly and succession of thermoacidic Fe(III)-oxide microbial mats.

## Materials and methods

### Site descriptions

**Figure 1:**
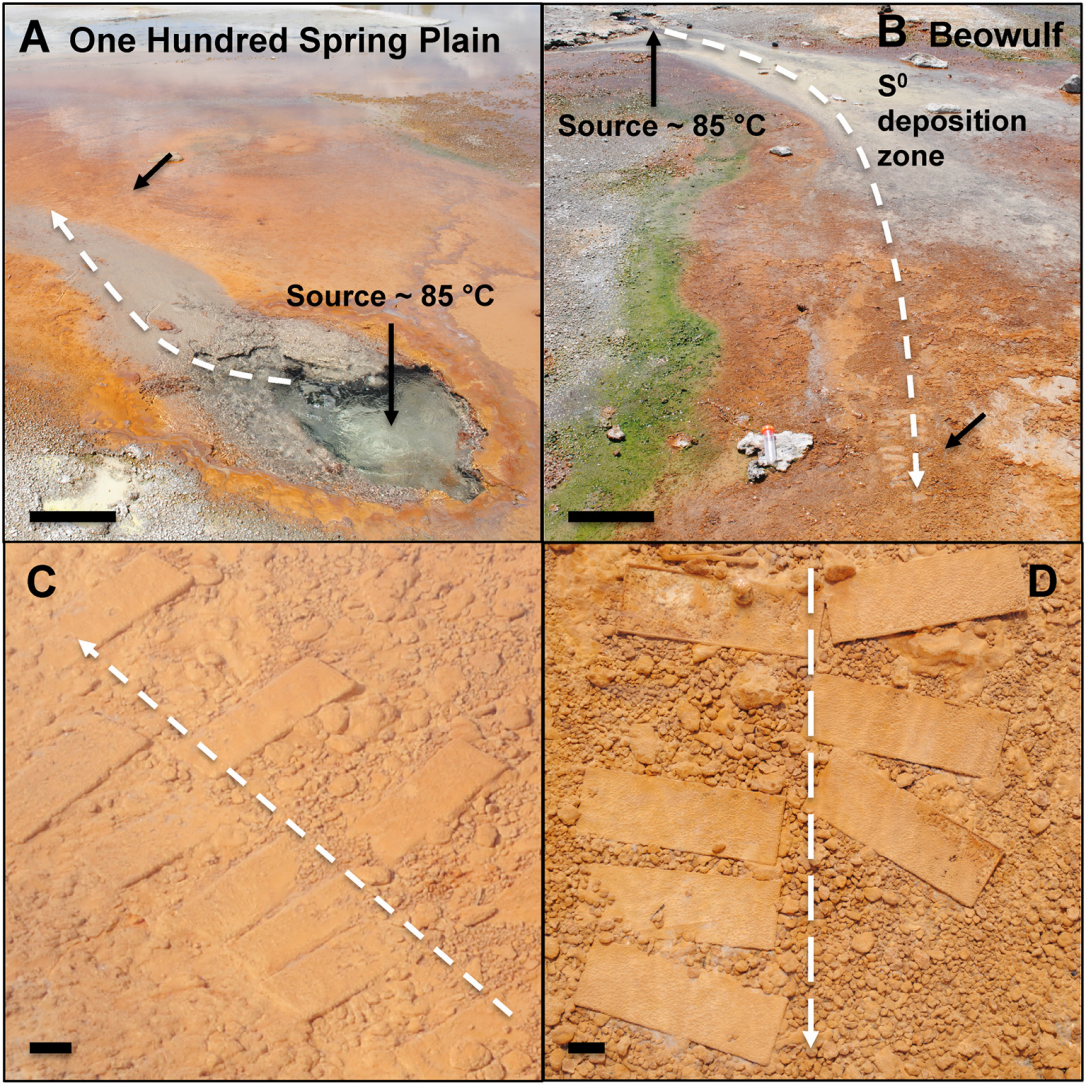
**Photographs of One Hundred Spring Plain (A) and Beowulf (B) Springs located in Norris Geyser Basin, Yellowstone National Park, WY, USA (scale bar = 30 cm)**. The black arrows represent the approximate location of slide placement in the primary outflow channel. Glass slides incubated for 70 days in OSP **(C)** and Beowulf **(D)** Springs (scale bar = 1 cm). The dashed white arrows represent direction of flow.

Two acidic geothermal springs in Norris Geyser Basin (NGB), YNP were chosen for this study based on long-term microbial and geochemical data obtained over the last 10–15 years (Jackson et al., 2001; Langner et al., 2001; Inskeep et al., 2004, 2005, 2010, 2013; Macur et al., 2004; Inskeep and McDermott, 2005; Ackerman, 2006; Kozubal et al., 2008, 2012, 2013; Beam et al., 2014; Jay et al., 2014; Jennings et al., 2014), which provides an excellent biological and physicochemical context for integrating results from the current study. Discharge source waters of “Beowulf” Spring (Thermal Inventory Number NHSP035; Lat/Lon = 44.731519, −110.711357) and an unnamed spring in One Hundred Spring Plain (OSP) (referred to here and in prior studies as OSP; Thermal Inventory Number NHSP115; Lat/Lon = 44.733044, −110.709012) exhibit low pH values (3–3.5), high temperatures (80–85°C) (Figure 1; Table 1), and contain reduced chemical species and high concentrations of dissolved inorganic carbon (DIC) formed by water-rock interactions occurring deep in the hydrothermal reservoir (Fournier, 1989). The concentrations of Fe(II), As(III), H_2_S, H_2_, and CH_4_ are significant and can potentially serve as electron donors for lithotrophic microbial populations (Macur et al., 2004; Inskeep et al., 2005; Kozubal et al., 2012). There is also an abundance of potential electron acceptors, which include O_2_, ${\text{SO}}_{4}^{2-}$, ${\text{NO}}_{3}^{-}$, Fe(III), and As(V) (Inskeep et al., 2005).

**Table 1:** **Physical and geochemical parameters measured over four field seasons corresponding to ***in situ*** slide incubations**.

**Spring (Position)**	**Temperature (°C)**	**pH**	**Channel velocity (cm s^−1^)**	**Reynolds number**	**O_2_ (aq)**	**Fe (TS)**	**As (TS)**	**CO_2_ (aq)**	**DOC**	**H_2_ (aq)**
					**μM**					**nM**
One Hundred Spring Plain (B)	72.8 (1.4)	3.5 (0.1)	2–5	1.5 • 10^3^	33 (11)	25 (7)	24 (6)	100 (40)	109 (55)	39 (40)
Beowulf (D)	67.6 (1.8)	2.9 (0.1)	20–30	1.4 • 10^4^	44 (6)	30 (3)	28 (4)	200 (180)	52 (12)	13 (7)

*Numbers in parentheses are equal to ± 1 standard deviation from the mean*.

*Reynolds Number = inertial forces/viscous forces, ρVD/μ: ρ = density of fluid (kg/m^−3^), V = fluid velocity (ms^−1^), D = channel depth (m), and μ = dynamic fluid viscosity [kg (ms)^−1^]. DOC, dissolved organic carbon*.

### Iron accretion rates

Iron-oxide accretion rates were measured *in situ* by inserting acid washed (2% HCl) and autoclaved borosilicate glass microscope slides (2.5 × 7.5 cm) into the main outflow channels of OSP and Beowulf Springs (Figure 1). Glass slides were chosen for the growth substrate for multiple reasons: (1) they mimic the native siliceous sinter that these iron oxide mats grow on, (2) they are easy to clean and sterilize, (3) glass is inexpensive, and (4) they are easy to deploy and sample in the hot spring study sites. Slides were inserted and removed at various time points during four field seasons (2010–2013) with the most extensive sampling occurring in 2012–2013 (Table S1). The number of slides inserted at any time point (~6–8) was limited to a small area in the outflow channel that exhibited the desired physicochemical environment (temperature range = 70–75°C in OSP Spring and 65–70°C in Beowulf Spring). Inclusion of two different geothermal springs provided a direct comparison of general trends in iron accretion and deposition. Iron oxides were removed from glass slides using a razor blade, dried overnight at 70°C, then transferred to 50 mL of 0.175 M ammonium oxalate buffer (pH = 3) to dissolve poorly-crystalline iron (oxyhydr)oxides (Loeppert and Inskeep, 1996). Slides without visible Fe(III)-oxide deposition were placed directly into the 0.175 M ammonium oxalate buffer. The extracting solutions were shaken (Model E600, Eberbach Co. Ann Arbor, MI, USA) for ~2 h to promote Fe(III)-oxide dissolution. All Fe-oxalate extractions were filtered (0.22 μm) into 15 mL Falcon™ tubes, and analyzed for Al, Ag, As, Ba, Be, B, Cd, Ca, Cr, Co, Cu, Fe, P, Pb, Mg, Mn, Mo, Na, Ni, K, S, Sb, Se, Si, Sr, Tl, Ti, V, W, and Zn using inductively coupled plasma-optical emission spectroscopy (OPTIMA 5300, Perkin-Elmer, Waltham, MA, USA). Rate constants of Fe(III)-oxide accretion were estimated in R using nonlinear least squares fits to the exponential growth rate equation x = x_o_e^kt^, where x = Fe accreted (μmol cm^−2^), x_o_ = initial Fe (μmol cm^−2^), k = first-order rate constant (day^−1^), and t = time (day). Different growth substrates including polyether ether ketone, titanium, polypropylene, polycarbonate, polytetrafluoroethylene, and ultra-high-molecular-weight polyethylene were also tested to determine if the rate of Fe(III)-oxide accretion on glass could also be observed on other substrates (substrates were deployed in Beowulf Spring from October 24 to November 6, 2013 (13 days) and total iron was measured as above (Figure S1).

### DNA extraction

DNA was extracted from slides grown over various time points to determine temporal microbial community composition. Biomass and mineralized iron oxides were removed from the slides by either scrapping off a known area with a sterile razor blade (when visible iron oxides were present), or by vortexing the slide in a 50 mL conical tube for ~30 s containing a sterile solution (autoclaved and 0.22 μm filtered) of 17.5 mM ammonium oxalate buffer (pH = 3), followed by cell collection on a 0.22 μm filter. The direct extraction method was performed when there were no visible Fe(III)-oxides present, which was common for slides incubated for <10 days. DNA was extracted from scraped Fe(III)-oxides or from cell-enriched filters using the FastDNA™ Spin Kit for Soil DNA extraction kit and protocol (MP Biomedicals, LLC, Solon, OH, USA). DNA was quantified using a Qubit® 2.0 fluorometer and Qubit® dsDNA High Sensitivity Assay Kit (range 0.2–100 ng total dsDNA) (Life Technologies Co.). DNA quantification (expressed as ng DNA cm^−2^ day^−1^) provided an estimate of biomass production as a function of time. Nonlinear model fits were generated in R as described above, where x and x_o_ are equal to DNA concentrations (ng cm^−2^).

### Scanning electron microscopy

Slides incubated *in situ* were also used for direct examination of microbial colonization with scanning electron microscopy. A subset of slides removed from the springs were fixed in 1% (final concentration) filter-sterilized (0.22 μm) glutaraldehyde. A Zeiss SUPRA 55VP field emission scanning electron microscope (Image and Chemical Analysis Laboratory, Montana State University) was used to image colonized slide surfaces, which were sputter-coated with iridium to minimize charging at low voltage (1 keV). Direct imaging of cells also provided estimates of *in situ* growth rates (3–5 random field views per estimate) on slides incubated for 4–15 days.

### Fluorescence *in situ* hybridization

Glass microscope slides with 0.3 cm^2^ round Teflon printed wells (SPI Supplies/Structure Probe, Inc. West Chester, PA, USA) were acid washed and autoclaved as above, incubated *in situ* for 6 days (October 24–30, 2013), removed and fixed with 1% paraformaldehyde (final concentration) for 5 min at 4°C. The fixative was removed from the slide by rinsing in a 1:1 solution of 1 X phosphate buffered saline (PBS): 100% ethanol (EtOH) and stored in a 50 mL canonical tube containing 1:1 1 X PBS: 100% EtOH at −20°C until hybridization.

Slides were dehydrated in an increasing ethanol series of 50, 80, and 100% for 3 min each, and then air-dried at room temperature. Hybridization buffer containing 40% formamide, 0.9 M NaCl, 20 mM Tris HCL, and 0.1% sodium dodecyl sulfate was added to multiple wells (30 μL) and 1 μL of each probe (6-FAM labeled Aqi338, Kubo et al., 2011; Cy5 labeled Arch915, Stahl and Amann, 1991) was added directly to the hybridization buffer on the wells (probe working solutions were 50 ng/μL for 6-FAM and 30 ng/μL for Cy5). The slide was placed in a 50 mL conical tube with tissue paper soaked in hybridization buffer (~1 mL) and incubated for 1.5 h in a 46°C hybridization oven. The slide was then washed for exactly 10 min in buffer that was pre-warmed to 48°C containing 46 mM NaCl, 20 mM Tris HCl, and 5 mM EDTA. The slide was then rinsed with room temperature distilled water and dried with laboratory air. The slide was then immediately visualized with a Leica SP5 inverted confocal scanning laser microscope (Leica Microsystems Inc., Buffalo Grove, IL, USA) at the Montana State University Center for Biofilm Engineering Confocal Microscopy Laboratory, or stored at −20°C for up to 2–3 days without fluorescence signal loss.

### Archaeal and bacterial 16S rRNA gene illumina sequencing and analysis

Archaeal and bacterial 16S rRNA gene sequences were amplified with the universal 515F (5′-GTG CCA GCM GCC GCG GTA A-3′)/806R (5′-GGA CTA CHV GGG TWT CTA AT-3′) (Caporaso et al., 2010) primer pair at the Department of Energy—Joint Genome Institute (Walnut Creek, CA, USA) and sequenced on an Illumina MiSeq (NCBI Bioproject ID PRJNA306640). A custom pipeline was utilized to screen Illumina Tag (iTag) 16S rRNA gene sequences (average length = 250 bp) with a database of relevant 16S rRNA gene sequences from YNP hot springs. Briefly, chimeras were removed from the iTag dataset, the sequences grouped into operational taxonomic units at 97% identity, then identified based on comparison to a curated group of long fragment (>1200 bp) 16S rRNA gene sequences from these and similar sites. Heatmaps showing the relative abundance of phylotypes were generated in R with the heatmap.plus package. Bray-Curtis dissimilarities were calculated with the vegan community ecology package in R (Oksanen et al., 2016). The abundance of phylotypes from mature Fe(III)-oxide mats were compared using Illumina 16S rRNA gene barcodes vs. Illumina random metagenome sequencing from matching samples. Barcoded 16S rRNA gene amplification using universal archaeal and bacterial primers (515F/806R; Caporaso et al., 2010; Earth Microbiome Project, http://www.earthmicrobiome.org/) on mature Fe(III)-oxide mats resulted in overestimation of *Hydrogenobaculum* spp. and underestimation of *M. yellowstonensis*-like organisms (see Table 2 in Results). The under- and overestimation of these phylotypes is caused by a mismatch of the 515F primer to the 16S rRNA gene in *M. yellowstonensis* (phylum Crenarchaeota), and is an important consideration for universal primer-based studies of Fe(III)-oxide microbial mats that contain abundant members of the Crenarchaeota. Although the relative abundance of *Hydrogenobaculum* spp. was corrected for two copies of the 16S rRNA gene, this adds more sequences to the PCR pool, and could result in additional overestimation. The 16S rRNA gene PCR amplification step using universal primers on these relatively simple communities illustrates how a single primer mismatch to the target sequence can cause a large discrepancy in relative abundance estimates of an important community member (i.e., *M. yellowstonensis*). Metagenome data for One Hundred Spring Plain (OSP_B) is located under the Integrated Microbial Genome Submission IDs 10386 (Illumina) and 1781 (454), and Beowulf Spring (BE_D) 10390 (Illumina), 2254 (454), and 278 (Sanger).

**Table 2:** **The relative abundance of different phylotypes from “mature” Fe(III)-oxide mats of One Hundred Spring Plain and Beowulf Spring (YNP) determined using random DNA sequencing over multiple years (and sequencing technologies), or short-fragment 16S rRNA gene sequencing (Illumina iTag)**.

**Spring**	**One Hundred Spring Plain Spring**			**Beowulf Spring**			
	**Random**	**Random**	**16S Tags**	**Random**	**Random**	**Random**	**16S Tags**
Sequencing technology	454	Illumina	Illumina	Sanger	454	Illumina	Illumina
pH/Temperature (°C)	3.6/72	3.5/75	3.5/75	3/65	2.9/66	2.9/68	2.9/68
**PHYLOTYPE**	**RELATIVE ABUNDANCE (%)**						
*M. yellowstonensis*	10.3	16	0.51	3.9	6.6	12	–
*Hydrogenobaculum* spp.	3.4	3	24.2	2	1.2	4.3	14
Geoarchaeota	43.4	33	41	1	3	6	4
Novel archaeal group 2	4	6	24	26	38	27	42
Thaumarchaeota	0.4	0.4	3	19	4.4	2	3.2
Novel archaeal group 3	0.1	0.12	–	4	3	5	26
Other Sulfolobales	2.5	4	3.3	3.9	10	11	8.5
Nanoarchaeota	0.2	0.6	–	0	0.1	0.6	–

*–, not identified, Sample dates for One Hundred Spring Plain 454 (July 15, 2010) and Illumina (October 11, 2011), and Beowulf Sanger (August 7, 2006), 454 (July 15, 2010), and Illumina (November 16, 2011)*.

### Oxygen microsensor measurements

Oxygen microsensor measurements were made on May 21, 2013 at OSP Spring Fe(III)-oxide microbial mats (temperature = 75°C, pH = 3.5, O_2_ (aq) = 55 μM) to identify variation in net areal O_2_ fluxes compared to prior measurements at OSP and Beowulf Spring Fe(III)-oxide mats (Bernstein et al., 2013). Custom Clark-type oxygen electrodes (tip diameter = 50 μm) designed with a high-temperature resistant electrolyte solution (Unisense A/S, Aarhus, Denmark) were used to make replicate measurements at OSP Spring (O_2_ microelectrode measurements were not repeated at Beowulf Spring Fe(III)-oxide mats due to difficulties (i.e., breaking microsensors) making these measurements on the harder Fe(III)-oxide mats present in this spring. Details on flux and reaction-diffusion modeling were discussed in Bernstein et al. (2013). Briefly, O_2_ microprofiles (*n* = 11) were modeled (first-order) using the dimensionless equation, u = (φ^2^ • ζ^2^)/2 − φ^2^ • ζ + 1, where u = ${\text{C}}_{{\text{O}}_{2}}$/${\text{C}}_{{\text{O}}_{2}}$', ζ = z/L_f_, and φ^2^ = k_1_${\text{L}}_{\text{f}}^{2}$/D_e_ (z = mat depth, ${\text{C}}_{{\text{O}}_{2}}$ = oxygen concentration, ${\text{C}}_{{\text{O}}_{2}}$' = bulk O_2_ concentration, k_1_ = first-order rate constant, L_f_ = mat thickness, and D_e_ = diffusion coefficient of O_2_). The solution of this equation provides an estimate of the relative contribution of the rate of O_2_ diffusion vs. the rate of O_2_ consumption (φ = Thiele Modulus), where φ > 0 indicates that diffusion is limiting and φ < 0 indicates that the reaction rate is limiting the observed rate of consumption (Thiele, 1939).

## Results

### Early colonization

**Figure 2:**
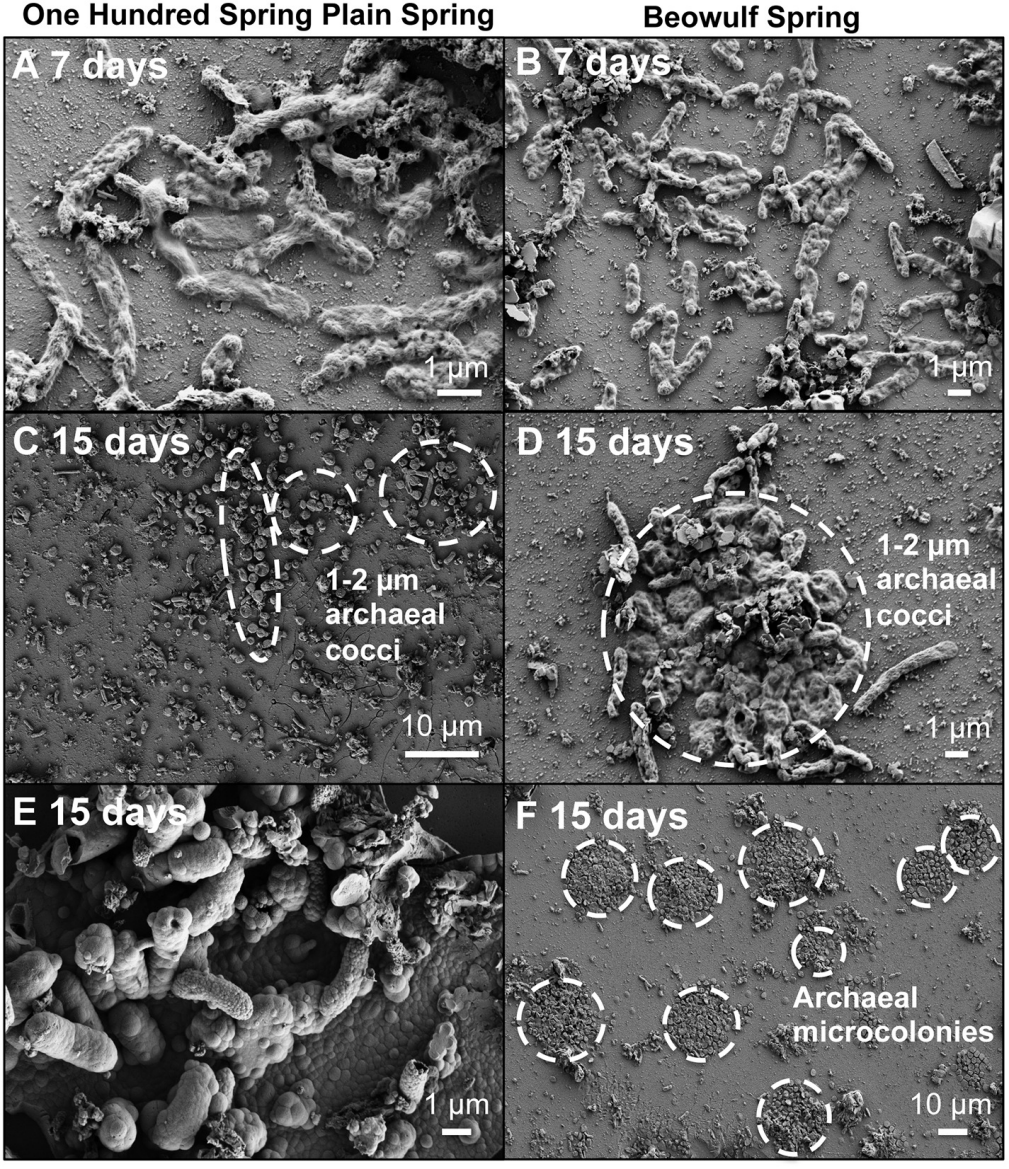
**Scanning electron micrographs of slides incubated for 7 and 15 days in Fe(III)-oxide mats from One Hundred Spring Plain (A,C,E from 7/13/2011 and 7/21/2013) and Beowulf Spring (B,D,F from 7/13/2011 and 7/21/2011)**. Archaeal microcolonies from Beowulf Spring **(F)** are highlighted with dashed circles.

**Figure 3:**
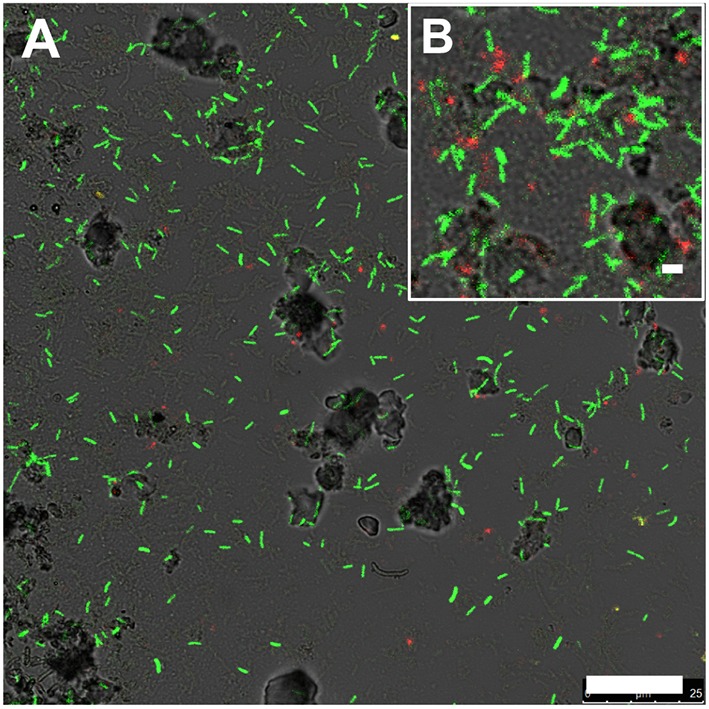
**Fluorescence ***in situ*** hybridization image of slides incubated in Beowulf Spring for 6 days (A,B)**. Green rods are *Hydrogenobaculum* spp. (6FAM-Aqi338) and red cocci are archaea (Cy5-Arch915). Scale bar = 20 μm **(A)** and 1 μm **(B)**.

Rod-shaped bacteria colonized slides rapidly, and significant cell densities of these organisms were observed using scanning electron microscopy (SEM) within 4–7 days of incubation in both OSP and Beowulf Springs (Figure 2). The taxonomic identity of these bacteria was confirmed to be *Hydrogenobaculum* spp. using 16S rRNA gene specific fluorescence *in situ* hybridization (FISH) probes (Figure 3). After confident taxonomic assignment of these bacteria, SEM images were useful for obtaining growth estimates during early incubation times (<14 days), prior to the extensive deposition of Fe-oxides and exogenous debris that precluded accurate cell counting. Colonization rate estimates of *Hydrogenobaculum* spp. were 3.7 ± 1.8 • 10^6^ and 6.8 ± 3.4 • 10^6^ cells cm^−2^ day^−1^ in OSP and Beowulf Springs, respectively. Coccus-shaped archaea were also identified at early time points (e.g., within 4–7 days) using both SEM and FISH (Figure 3), but were considerably less abundant than *Hydrogenobaculum* spp. (Figures 2, 3). Although *M. yellowstonensis* probes have proven difficult in Fe(III)-oxide samples *in situ* (Kozubal et al., 2008), archaeal probes were positive and molecular data (below) indicated that early-colonizing archaea were *M. yellowstonensis*-like organisms. Early-colonizing archaea were often found as individuals within 4–6 days, and as microcolonies containing up to 50 cells within 15 days (Figures 2C–E). These cocci colonized at rates of approximately 9.2 ± 5.1 • 10^5^ and 8.6 ± 4.1 • 10^5^ cells cm^−2^ day^−1^ in OSP and Beowulf Springs, respectively, which is nearly five times slower than the colonization rates by *Hydrogenobaculum* spp. Visible Fe-oxide staining from 7 to 14 days also corresponded to the detection and proliferation of these archaea. *Hydrogenobaculum* spp. and *M. yellowstonensis* were often observed in close spatial proximity (Figure 2D), which suggests that these populations are interacting *in situ*.

### Iron oxide accretion

The biomineralization of poorly-crystalline Fe(III)-oxide phases occurred as visible crusts on *Hydrogenobaculum* rods and filaments at times >7 days (Figure 2). These arsenate-rich, poorly-crystalline Fe(III)-oxide phases (Inskeep et al., 2004) begin to dominate the available surface area and form larger (>1 μm) crusts on outer cell surfaces of *Hydrogenobaculum* spp. and other inorganic templates (e.g., SiO_2_ and alunite) at incubation times greater than 14 days (Figure 2E). Direct observations of temporal changes in Fe(III)-oxide deposition using SEM were corroborated with data obtained on Fe(III)-oxide accretion as a function of time.

**Figure 4:**
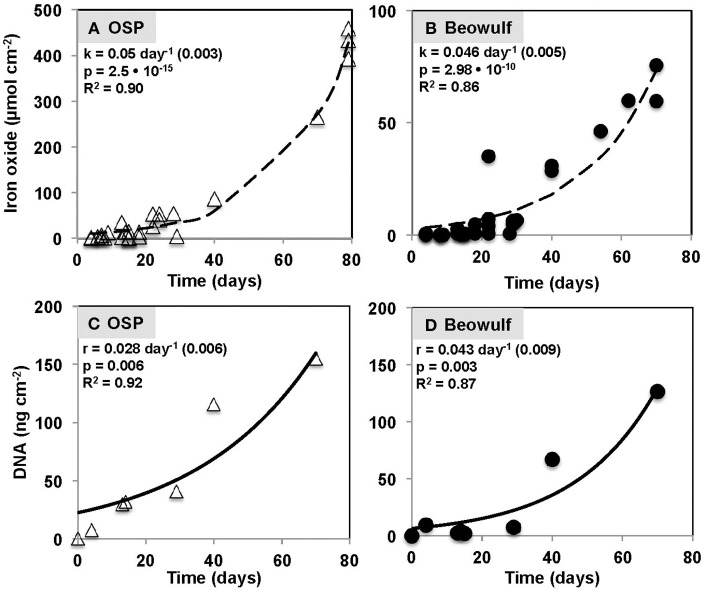
**Iron oxide accretion measured on glass slides incubated as a function of time in One Hundred Spring Plain (OSP) (A) and Beowulf (B) Springs outflow channels in Norris Geyser Basin, Yellowstone National Park**. The dashed black line represents a model fit to the exponential rate expression Fe_x_ = Fe_o_e^kt^, where Fe_x_ is iron oxide accreted (μmol Fe cm^−2^), Fe_o_ is the initial iron oxide concentration (μmol Fe cm^−2^), k is the first-order rate constant (day^−1^), and t is time (day). Total DNA concentrations determined from slides incubated in One Hundred Spring Plain (OSP) **(C)** and Beowulf Spring **(D)** over a time period of 70 days. The solid black line represents a model fit to the exponential rate expression DNA_x_ = DNA_o_e^rt^, where DNA_x_ is DNA accumulated (ng DNA cm^−2^), DNA_o_ is the initial DNA concentration (ng DNA cm^−2^), r is the first-order rate constant (day^−1^), and t is time (day).

Iron oxide deposition increased exponentially with time (days) in both OSP and Beowulf Springs (Figures 4A,B), and was modeled using a first-order rate equation (Fe_x_ = Fe_o_e^kt^) where Fe_x_ is Fe(III)-oxide accreted (μmol Fe cm^−2^), Fe_o_ is the initial Fe(III)-oxide concentration (μmol Fe cm^−2^), and k is the empirical first-order rate constant (day^−1^). A lag phase of Fe(III)-oxide accretion occurred from 0 to ~30 days (Figures 4A,B), which corresponded to the slower growth rate of Fe(II)-oxidizing microorganisms. The fitted first-order rate constants for Fe(III)-oxide accretion were 0.05 day^−1^ (std. error = 0.003, *p* = 2.5 • 10^−15^) and 0.047 day^−1^ (std. error = 0.005, *p* = 2.98 • 10^−10^) at OSP and Beowulf, respectively. Similar rate constants (within 10%) describing the accumulation of Fe-oxides were observed for OSP and Beowulf springs across multiple field seasons and suggest that similar processes control the deposition of Fe(III)-oxides in these habitats. The lower amount of Fe(III)-oxide accreted by 70 days of incubation in Beowulf Spring relative to OSP was significant (*p* = 0.0018, student's two-tailed *T*-test) and may be attributed to differences in spring geochemistry (e.g., lower pH and temperature) and local hydrodynamic conditions. Specifically, the higher flow velocities and higher Reynolds numbers in Beowulf vs. OSP Spring (*R* = 1.4 • 10^4^ and 1.5 • 10^3^, respectively) resulted in lower amounts of Fe deposition (Table 1). The vertical growth of Fe(III)-oxide mats in acidic geothermal springs occurred at a rate of ~15–30 μm day^−1^ (0.5–1 mm month^−1^), although the maximum vertical growth is ultimately limited by channel water depths. Maximum rates of Fe(III)-oxide accretion in acidic geothermal springs (~0.9 μmol Fe cm^−2^ day^−1^) fall within the range observed for other systems (0.09–9 μmol Fe cm^−2^ day^−1^), which includes estimates of Fe deposition rates in banded iron formations (Konhauser, 1998) as well as observations in circumneutral pH environments (Hanert, 1974; Emerson and Revsbech, 1994).

### Temporal changes in microbial community composition

**Figure 5:**
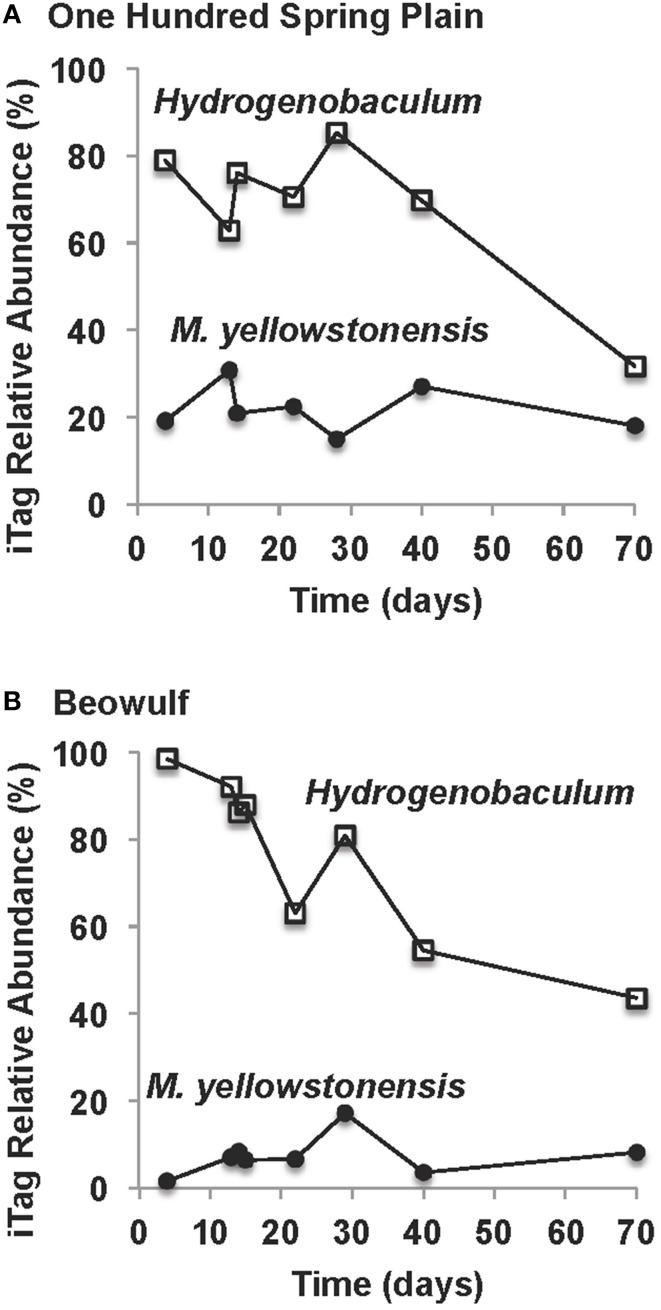
**Relative abundance (16S rRNA gene iTags) of key lithoautotrophic microbial populations over the time course of slide incubations in One Hundred Spring Plain (A) and Beowulf (B) Springs (***Hydrogenobaculum*** spp. = open squares; ***Metallosphaera yellowstonensis*** = closed black circles)**.

The amount of microbial biomass (expressed as ng DNA cm^−2^) increased as a function of time in OSP and Beowulf Springs, and also followed a first-order rate equation (Figures 4C,D), similar to Fe(III)-oxide accretion. A lag phase in the accumulation of total community DNA was also observed up to ~30 days, after which DNA increased exponentially. The fitted rate constants for DNA accumulation were 0.028 day^−1^ (std. error = 0.0063, *p* = 0.0062) and 0.043 day^−1^ (std. error = 0.0087; *p* = 0.0026) for OSP and Beowulf Springs, respectively. These values are essentially similar to the fitted rate constants for Fe accretion, and it is clear from the time series behavior that Fe accumulation and DNA accumulation are also correlated (Figure 4). Molecular data confirmed that the lithoautotrophic populations of *Hydrogenobaculum* spp. and *Metallosphaera yellowstonensis* were the dominant community members (>90%) at early stages of mat development (<30 days) (Figure 5). The abundance of *M. yellowstonensis* was relatively constant (10–20%) throughout mat development. In contrast, *Hydrogenobaculum* spp. abundance was highest during early stages of colonization, and declined after 15–30 days (Figure 5) as mat depth increased and other heterotrophic archaea contributed to the total mat community.

**Figure 6:**
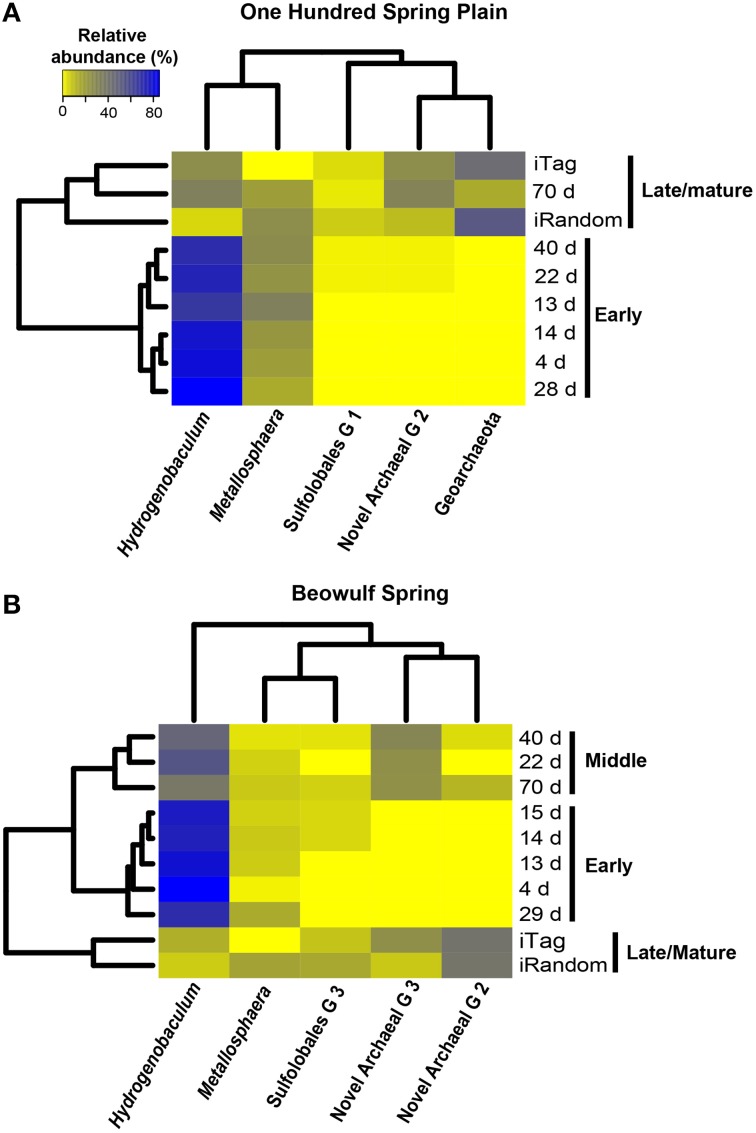
**Relative abundance (16S rRNA gene Illumina barcodes) of different phylotypes determined on slides incubated in One Hundred Spring Plain (A) and Beowulf (B) Springs from 4 to 70 days**. The dendrograms on the y and x axes represent the Bray-Curtis dissimilarity matrix between taxon abundance at different time points and taxon abundances, respectively.

Dendrograms of microbial community structure as a function of time were compared to population abundances of mature Fe(III)-oxide mats (Figure 6). The population abundances of Fe(III)-oxide mat communities from early incubation times (<40 days) were more similar to one another compared to later time points (>70 days), which reflects progression toward “mature” 0.5–2 cm thick Fe(III)-oxide mats (Table 2, Figure 6). The dominant microbial population observed from 4 to 70 days was *Hydrogenobaculum* spp., which was consistent with direct observations using SEM and FISH. Although *Hydrogenobaculum* spp. were especially dominant at times <14 days (Figure 6), their abundance declined on average of ~1% per day with increasing mat depths. Using this estimate, relative abundances of *Hydrogenobaculum* spp. would reach ~1–3% after 100 days, which is within the range observed for “mature” Fe(III)-oxide mats of 0.5–2 cm thickness (Table 2). *Metallosphaera yellowstonensis* was the only other population detected in significant numbers at early time points and remained relatively constant over the time series, representing ~10–20% of the total microbial community (Figure 6). Iron-oxide accretion increased significantly after 40 days and this corresponded to the detection of several heterotrophic archaea, which represented the majority of the total community (in aggregate) by 70 days (Table 2, Figure 6), as well as in “mature” Fe-oxide mats (reaching depths of 0.5–2 cm).

Although the predominant lithoautotrophs responsible for Fe(III)-oxide mat formation are the same in the two geothermal springs studied, different heterotrophic assemblages between sites were also evident. For example, populations of a novel archaeal Group 3 (NAG3), which are phylogenetically related to the archaeal order Thermoplasmatales (Inskeep et al., 2010; Kozubal et al., 2012), were especially important in Beowulf Spring at later time points (Figure 6). Furthermore, members of Sulfolobales Group 3, which includes a novel heterotrophic Fe(II)-oxidizing species (Kozubal et al., 2012), appeared later in community succession (~20 days) at Beowulf Spring, and may contribute to Fe(III)-oxide accretion in this site. Several other heterotrophic archaea were observed in mid to later stages of Fe(III)-oxide mat succession, and although these phylotypes were present in lower abundance (<1–2%), all have been observed in acidic Fe(III)-oxide mats from YNP (Kozubal et al., 2012), and these chemoorganotrophs do not have genes required for the fixation of CO_2_ (Jennings et al., in review), which suggests that they rely (in part) on organic C produced by *Hydrogenobaculum* spp. and *M. yellowstonensis*.

The relative abundance of different phylotypes in “mature” (0.5–2 cm thick) Fe(III)-oxide microbial mats was estimated from random shotgun metagenome and 16S rRNA gene sequencing (Table 2, Figure 6). *Metallosphaera yellowstonensis* populations ranged from ~4 to 16% of the microbial community across temperatures of 65–75°C (Table 2), and is consistent with the reported optimum growth temperature (Kozubal et al., 2008). Other Sulfolobales populations were also detected, and a representative of Sulfolobales Group 3 (strain MK5) has been shown to oxidize Fe(II) heterotrophically (Kozubal et al., 2012). *Hydrogenobaculum* spp. were less abundant in mature Fe(III)-oxide mats (Table 2), which is consistent with the measured decline in these populations over time (Figures 5, 6). Members of the Geoarchaeota (Kozubal et al., 2013) represent the dominant heterotrophic phylotype in OSP Spring, whereas members of a novel archaeal Group 2 (NAG2; Kozubal et al., 2012) were the dominant heterotrophs in Beowulf Spring. Deeply-branching Thaumarchaeota (Beam et al., 2014) were also observed in mature Fe mats, and were abundant in Beowulf Spring (Table 2). Differences in temperature and pH between the two sites likely influence the relative abundance(s) of these heterotrophic archaeal populations (Kozubal et al., 2012).

### *In situ* oxygen consumption and formation of microterracettes

**Figure 7:**
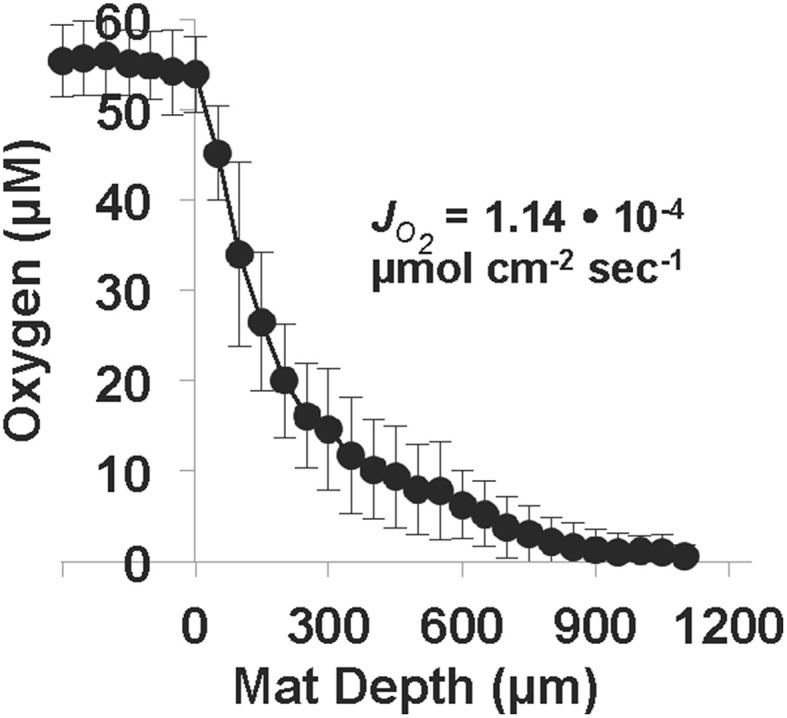
**Oxygen microprofiles (***n*** = 11, electrode tip diameter = 50 μm) measured in One Hundred Spring Plain Fe(III)-oxide mats (May 2013; temperature = 75°C, pH = 3.5, O_**2**_ (aq) ~55 μM)**. The diffusive flux of oxygen, $J_{{\text{O}}_{2}}$, was estimated from the measured concentration profiles as a function of Fe(III)-oxide mat depth. Position zero refers to the aqueous-Fe(III)-oxide mat interface.

Oxygen microelectrode measurements in OSP Spring Fe(III)-oxide mats revealed an areal O_2_(aq) consumption rate of 1.14 • 10^−4^ μmol cm^−2^ s^−1^ (Figure 7). The concentration of O_2_(aq) ranged from ~55 μM at the mat-aqueous interface and dropped to below detection (<0.3 μM) within the top 1 mm. These observations are consistent with prior O_2_ microelectrode measurements in the same Fe(III)-oxide mat systems (Table 3). Dimensionless reaction-diffusion model fits (see Materials and Methods) of oxygen microprofiles resulted in an estimate of 28 for the Thiele modulus (φ), which indicates that the rate of biotic O_2_ consumption was at least an order of magnitude faster than O_2_ diffusion into the Fe(III)-oxide mats (Bernstein et al., 2013). Thus, the microbial consumption of O_2_ is limited by the rate of O_2_ diffusion; an average flux of O_2_ into these poorly-crystalline Fe(III)-oxide mats was 1.2 ± 0.5 • 10^−4^ μmol cm^−2^ s^−1^ at temperatures ranging from 60 to 75°C (Table 3).

**Table 3:** **Oxygen flux estimates, penetration depths, and Thiele^a^ moduli describing oxygen diffusion in thermoacidic Fe(III)-oxide mats from Norris Geyser Basin, Yellowstone National Park**.

**Spring/Temperature (°C)**	**Sample date**	**Net areal O_2_ flux (μmol cm^−2^ s^−1^)**	**Penetration depth (μm)**	**Thiele modulus**	**References**
One Hundred Spring Plain/75	May 21, 2013	1.14 • 10^−4^	950	28	This study
One Hundred Spring Plain/75	August 18, 2010	1.41 • 10^−4^	750	30	Bernstein et al., 2013
Beowulf/68	July 6, 2011	1.64 • 10^−4^	250–1000	60	Bernstein et al., 2013
Beowulf/60	July 13, 2004	5 • 10^−5^	500	nd	Kühl and Kozubal, unpublished

a *see Methods for definition of Thiele modulus*.

**Figure 8:**
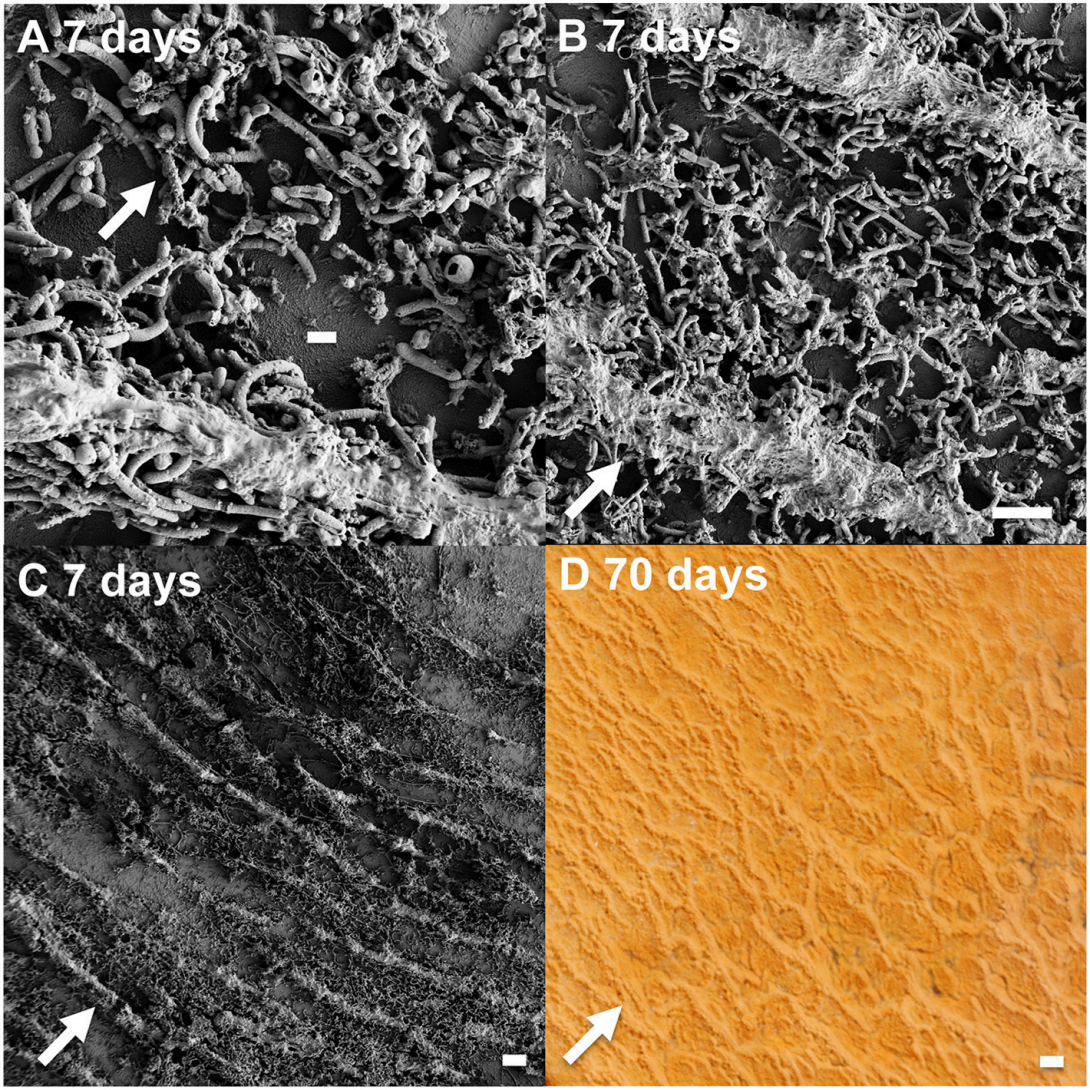
**Growth and formation of microterracettes from slides incubated in Beowulf Spring**. Scanning electron micrographs **(A–C)** of ridge structures: scale bar = 1 μm **(A)**, and 10 μm **(B,C)**. Photograph of larger, 1 mm ridges **(D)** present in 70 days Fe(III)-oxide mats incubated for 70 days (scale bar = 1 mm). The flow direction (arrows) is perpendicular to microterracettes.

Microterracettes containing both *Hydrogenobaculum* spp. and *M. yellowstonensis* were observed within 6 days, already reaching heights up to 10 μm and spaced at ~10–20 μm intervals (Figure 8). These smaller microterracettes may coalesce to form larger (~1 mm high) structures (Figure 8), which evolve into visible cm-scale microterracettes common in mature Fe(III)-oxide mats (Figure 1). These structures increase the overall surface area and flux of O_2_ into the mat system, similar to the formation of “wrinkles” in microbial communities, which have been shown to form as a result of O_2_ mass transfer limitations to microorganisms (Okegbe et al., 2014). The relative size and periodicity of microterracettes is likely correlated with the extent and size of the mass transfer boundary layer, which is a function of water velocity and character (i.e., turbulent vs. laminar flow), as well as physical properties of the biomineralized mat.

### Vertical stratification of microbial populations

**Figure 9:**
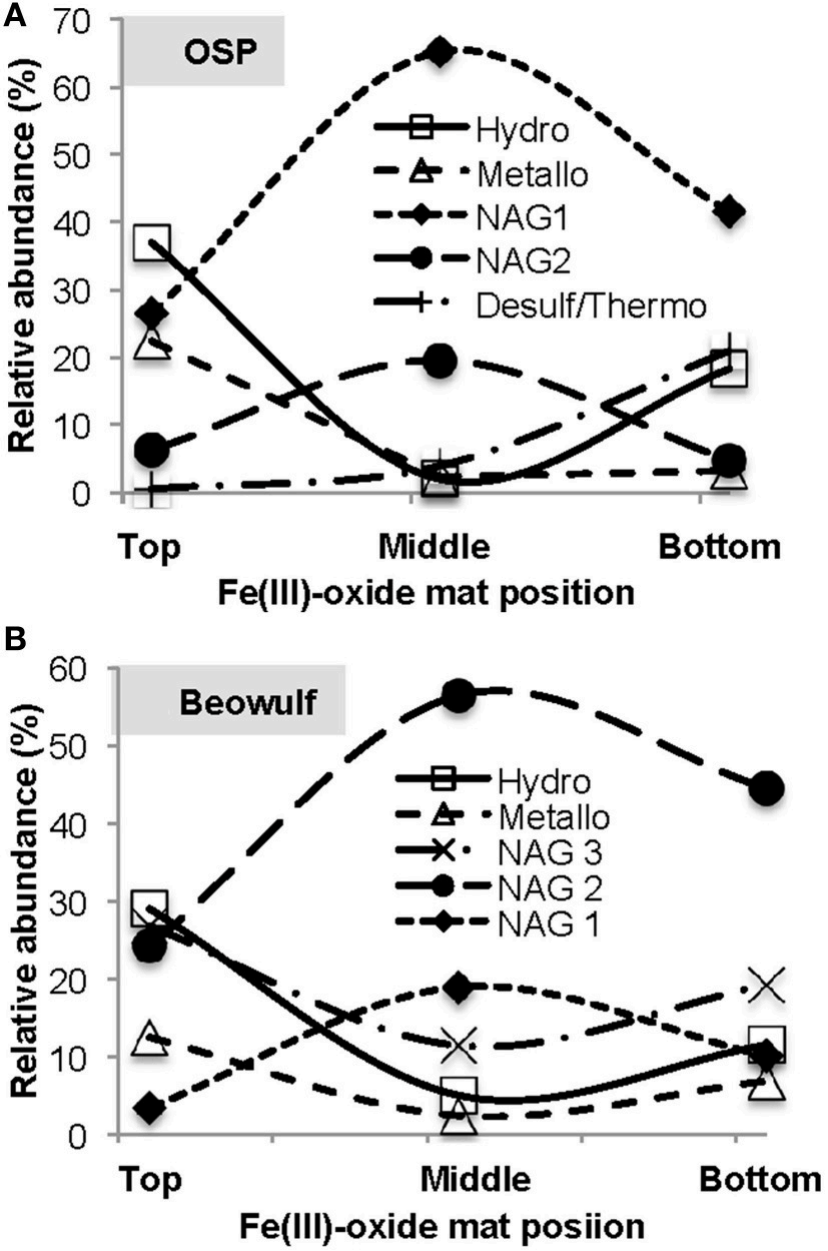
**Relative abundance of taxa (16S rRNA gene Illumina barcodes) as a function of depth in mature thermoacidic iron oxide mats from One Hundred Spring Plain (A) and Beowulf (B) Springs**. The top position refers to the upper 1 mm oxygenated zone, the middle position represents depths of ~2–7 mm, and the bottom position represents hypoxic regions > 10 mm.

Oxygen gradients result in the stratification of Fe(III)-oxide mat community members. We dissected “mature” Fe(III)-oxide mats into discrete zones (top 1 mm, middle, bottom) for 16S rRNA gene (iTag) analysis, which revealed stratification of the microbial community as a function of mat depth (Figure 9). Specifically, *M. yellowstonensis* and *Hydrogenobaculum* spp. were more abundant in the top 1 mm of the Fe(III)-oxide surface relative to middle or bottom positions, which is consistent with the high O_2_ requirements of these lithoautotrophs. Microaerobic chemoorganotrophs (i.e., NAG2 and Geoarchaeota) were more abundant in middle and bottom mat sections (Kozubal et al., 2012). The relative abundance of hypoxic community members such as *Acidilobus* spp. (order Desulfurococcales) increased in bottom mat positions, which is consistent with the metabolic attributes of these organisms (Jay et al., 2014) and the low O_2_ concentrations (<0.3 μM) observed at mat depths >1 mm (e.g., Table 3, Figure 8). These data are also consistent with prior results showing that active Fe(II)-oxidizing *M. yellowstonensis* populations are higher in the upper (~1 mm) mat layer (Bernstein et al., 2013).

### Adsorption of oxyanions

**Figure 10:**
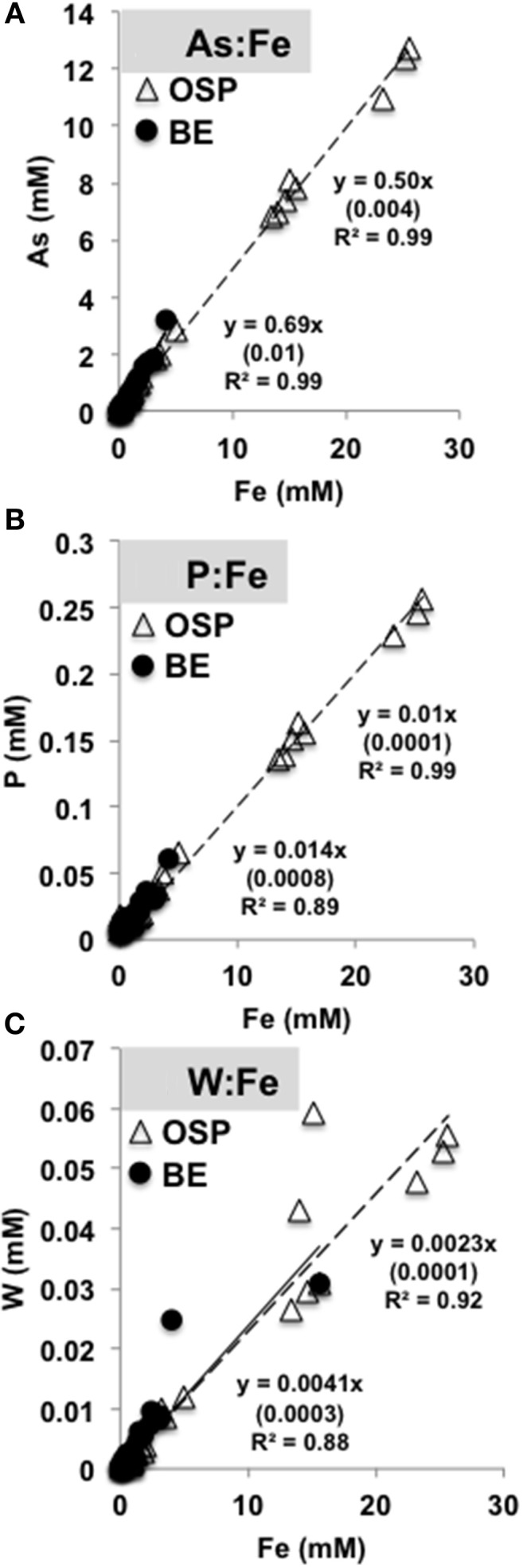
**Molar ratios of arsenic (A), phosphorous (B), and tungsten (C) to total solid phase iron extracted from slides incubated in One Hundred Spring Plain (open triangles, dotted black line) and Beowulf (filled circles, black line) Springs (Figure 1)**. Numbers in parentheses are the standard error.

The biomineralization of Fe(III)-oxides in acidic mats of NGB results in the concomitant adsorption, coprecipitation and/or biomass uptake of oxyanions such as arsenate, phosphate and tungstate over time (Figure 10) (e.g., Leblanc et al., 1996; Karl et al., 1988). Molar ratios of As:Fe over all time points ranged from 0.5 (OSP) to 0.67 (Beowulf), which is consistent with prior measurements of As:Fe ratios in “mature” Fe(III)-oxide mats (Langner et al., 2001; Inskeep et al., 2004; Macur et al., 2004). The co-accumulation of As, P, W and Fe could provide evidence of microbiological activity under acidic conditions [e.g., Fe(II) and As(III) oxidation and subsequent biomineralization]. Molar ratios of P (0.01) and W (0.002) to Fe were similar between OSP and Beowulf Springs (Figure 10). Tungsten may substitute for molybdenum in enzymes (e.g., dimethyl sulfoxide molybdopterins) utilized by thermoacidophilic organisms to perform specific functions such as arsenite oxidation or degradation of organic matter (Kletzin and Adams, 1996).

## Discussion

**Figure 11:**
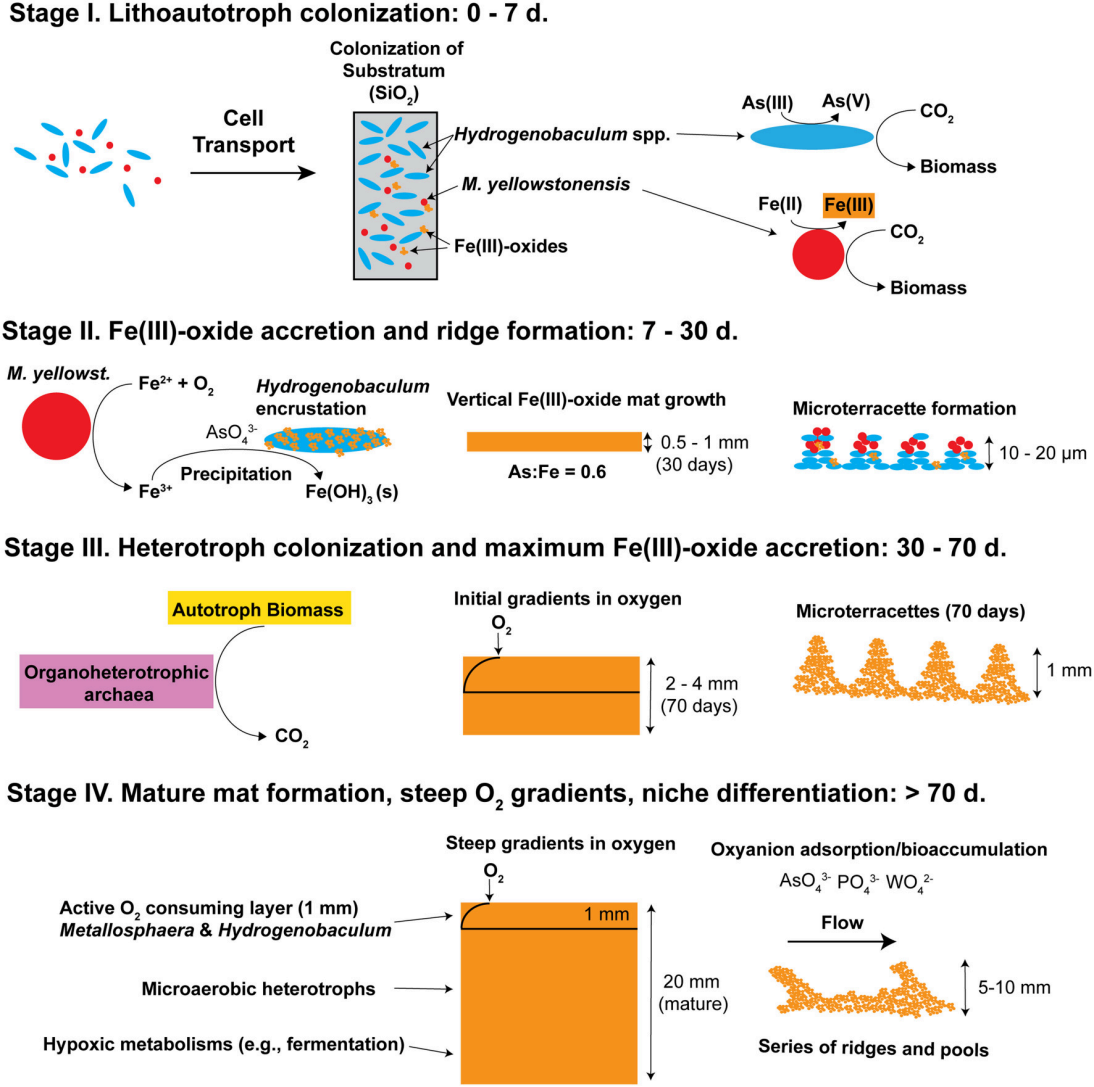
**Conceptual model of iron oxide mat development (four stages) in high-temperature, acidic geothermal springs of Yellowstone National Park**. Stage I is defined by the primary colonization of lithoautotrophic populations of *Hydrogenobaculum* spp. and *Metallosphaera yellowstonensis*. Stage II is represented by visible iron oxide accretion, encrustation of *Hydrogenobaculum* spp. by Fe(III)-oxides, and initial formation of microterracettes. Heterotrophic archaea begin colonizing in Stage III and gradients in O_2_ are apparent. As iron oxide depth increases to form “mature” 0.5–2 cm thick mats (Stage IV) the microbial community undergoes succession toward organoheterotrophic archaea, where steep gradients in oxygen, and macroscale ridges and pools are observed.

A conceptual model describing the assembly and succession of thermoacidic Fe(III)-oxide microbial mats was developed by integrating geochemical, imaging, and molecular measurements across multiple scales of observation. The life cycle of a high-temperature Fe(III)-oxide mat in the acid-sulfate-chloride springs of Norris Geyser Basin (YNP) can be represented by four stages (Figure 11). Early colonization (Stage I) by aerobic, chemolithoautotrophic populations of *Hydrogenobaculum* spp. and *Metallosphaera yellowstonensis* provides critical founder populations for initial surface roughness and continued mat growth. *Hydrogenobaculum* spp. exhibit significantly greater colonization rates than *M. yellowstonensis* (factor of 5) in high-temperature acidic Fe(III)-oxide mats. The aerobic oxidation of arsenite (and/or reduced sulfur species) by *Hydrogenobaculum* spp. (D'Imperio et al., 2007, 2008; Hamamura et al., 2009) and Fe(II) by *M. yellowstonensis* (Kozubal et al., 2008) is coupled with the fixation of DIC as a primary C source via the reductive tricarboxylic acid and 3-hydroxypropionate/4-hydroxybutyrate cycles, respectively (Berg et al., 2007; Takacs-Vesbach et al., 2013; Jennings et al., 2014), and provides the Fe(III) and As(V) necessary for the formation of high-As, poorly-crystalline Fe(III)-oxides characteristic of these springs (Langner et al., 2001; Inskeep et al., 2004; Macur et al., 2004).

Appreciable amounts of Fe(III)-oxides are produced between ~7 and 30 days (Stage II), which correlates with the establishment of *M. yellowstonensis* (e.g., microcolonies of 10–50 cells), and which results in the encrustation of rapidly-growing *Hydrogenobaculum* rods and filaments (Figure 2). The cell surface of *Hydrogenobaculum* may contain macromolecules that aid in the nucleation of poorly-crystalline Fe(III)-oxide phases, because these cells are encrusted preferentially (also see Macur et al., 2004). Moreover, Fe(III)-oxides do not accumulate on the cell surface(s) of *M. yellowstonensis* under culture conditions (Kozubal et al., 2008), or *in situ* (Figure 2). *Hydrogenobaculum* spp. inhabit a large reach (several meters) within the outflow channels of OSP and Beowulf Springs (Figures 1A,B), and data obtained during the first 4 days of slide placement suggest that these organisms are continually colonizing the upper surface of iron mats where O_2_ concentrations are highest. Higher rates of cell growth occur along microscale ridges as early as 6–7 days (Figure 8). These structures reflect the microbial control of microterracette formation in acidic Fe(III)-oxide mats, and are likely formed in response to O_2_ mass transfer limitations (Figure 7) to growing chemolithotrophic populations (i.e., *Hydrogenobaculum* and *M. yellowstonensis*). Cell growth proceeds upward into the shallow, high-velocity (~20–30 cm s^−1^) aqueous phase where O_2_(aq) concentrations range from 20 to 60 μM (Table 1), and results in Fe(III)-oxide mat heights of 0.5–1 mm within 30 days.

Continued Fe(III)-oxide accretion and cell growth >30 days results in significant accumulation of biomass C and niche diversity (e.g., O_2_ gradients) that allow for the colonization of heterotrophic archaea (Stage III). Moreover, the probability of trapping exogenous detritus and/or debris from landscape sources increases as the surface roughness increases due to cell growth and Fe(III)-oxide formation. For example, plant material, diatoms, and wind-blown solid-phases can be trapped by the complex series of ridges and valleys established during mat development. The later succession of heterotrophs also suggests that they require specific metabolites and/or cofactors produced by autotrophic populations. The primary archaeal organoheterotrophs observed from 30 to 70 days using iTag analysis (16S rRNA) included members of the candidate phylum Geoarchaeota (Kozubal et al., 2013), novel archaeal Groups 2 and 3 (Inskeep et al., 2010, 2013; Kozubal et al., 2012) and members from two additional Sulfolobales lineages, which have also been observed at these lower abundances (1–2%) in prior Fe-mat studies (Inskeep et al., 2010; Kozubal et al., 2012). By 70 days, Fe(III)-oxide mat depths can reach 2–4 mm, and have developed initial O_2_ concentration gradients (Figure 7) that support further niche diversification to include hypoxic populations. Although the fixation of CO_2_ by autotrophic *Hydrogenobaculum* spp. and *M. yellowstonensis* populations represents a significant fraction of the biomass C in “mature” Fe(III)-oxide mats (no less than ~40% DIC signature; Jennings et al., 2014), the relative abundance of *Hydrogenobaculum* spp. is actually negatively correlated with Fe(III)-oxide accretion over time (Figures 5, 6) due to the rise of other heterotrophic populations; although *Hydrogenobaculum* spp. are constantly colonizing and growing in the active O_2_ consuming layer (i.e., the upper 1 mm of mat) (Figure 3).

Ultimately, the shallow water depth in the outflow channels (<2 cm) becomes a limiting factor for the maximum thickness of these Fe(III)-oxide mats (Stage IV). Several metagenomes were obtained from “mature” Fe(III)-oxide mats of this thickness in both spring positions used in the current study, and these samples were also analyzed using iTags (Table 2). The integration and comparison of these datasets with the temporal incubation studies provides an opportunity to understand the cycle of Fe(III)-oxide mat development. Larger microterracettes (~1 mm), which are visible even after ~70 days (Figure 8), continue to grow to form a series of centimeter-scale terracettes over a temperature range of ~55–75°C (Figure 1). These thicker Fe(III)-oxide terracettes reveal changes in population abundance as a function of mat depth (Figure 9) that are consistent with expected gradients in O_2_ observed in “mature” mats (Figure 7). The succession of early colonizing autotrophs to later colonizing heterotrophs is directly applicable to other Fe(III)-oxide mat hot spring ecosystems; autotroph-heterotroph successional patterns are a common theme in other microbial mat communities, as well as larger-scale ecosystems (Odum, 1969).

High-temperature Fe(III)-oxide mats of Norris Geyser Basin are modern-day stromatolites (Riding, 1999), which form as a direct consequence of microbial metabolism and associated hydrogeochemical controls. The preferential biomineralization of Fe(III)-oxides on cell surfaces of *Hydrogenobaculum* spp. may be related to enhanced nucleation rates of poorly-crystalline Fe(III)-oxide phases. Other members of the Aquificales are also thought to promote the nucleation and growth of aragonite (CaCO_3_; Kandianis et al., 2008; Fouke, 2011). Stable carbon isotopes (i.e., ^13^C) also provide signatures indicative of biological activity, such as evidence for significant CO_2_ fixation in extant Fe(III)-oxide mats (Jennings et al., 2014). Micro-morphological changes across different stages of Fe(III)-oxide mat development including cell encrustation by Fe(III)-oxides and the formation of microterracettes also reveal microbiological signatures that may provide a reference for comparison to other extant and ancient Fe(III)-oxide hot spring ecosystems on Earth, or Fe(III)-oxide mineral deposits identified on other planetary systems such as Mars (Madden et al., 2004).

## Author contributions

JB and WI conceived and designed experiments. JB, HB, ZJ, MK, RJ, and WI performed experiments. ST contributed Illumina sequencing and support. JB, HB, ZJ, and WI analyzed data. JB and WI wrote the manuscript.

### Conflict of interest statement

The authors declare that the research was conducted in the absence of any commercial or financial relationships that could be construed as a potential conflict of interest.
