# Supplementary material for: Assembly and Succession of Iron Oxide Microbial Mat Communities in Acidic Geothermal Springs
Source: Front Microbiol. 2016 Feb 15;7:25. doi: 10.3389/fmicb.2016.00025 (PMC4753309; doi:10.3389/fmicb.2016.00025)
Supplement: Supplementary file 1 [file Table1.PDF]

Table S1. Summary of all sampling dates and iron oxide accretion data. DNA extraction was preformed on sample rows highlighted in grey.

OSP Spring			Beowulf Spring		
[1]Date (d/m/y)	Days	Fe (umol cm^{-2})	[1]Date (d/m/y)	Days	Fe (umol cm^{-2})
3-Nov-2012	4	2.37	3-Nov-2012	4	0.76
3-May-2013	4	0.06	3-Nov-2012	4	0.39
30-Oct-2013	6	2.96	3-May-2013	4	0.03
13-Jul-2011	7	0.89	28-Aug-2013	8	0.06
6-Nov-2013	7	6.83	28-Aug-2013	8	0.02
7-May-2013	8	0.37	7-May-2013	8	0.06
15-Nov-2013	9	12.54	15-Nov-2013	9	0.01
3-Oct-2012	13	1.85	3-Oct-2012	13	0.67
6-Nov-2013	13	34.71	11-Jul-2013	13	0.61
26-Jul-2012	14	12.87	16-Nov-2013	13	2.74
21-Jul-2011	15	14.56	26-Jul-2012	14	0.25
11-Jul-2013	15	0.16	21-Jul-2012	15	0.31
11-Jul-2013	15	0.31	21-Jul-2011	15	0.62
12-Jul-2012	15	1.24	12-Jul-2012	15	0.34
21-May-2013	18	13.13	29-Jul-2013	18	0.79
29-Jul-2013	18	2.94	21-May-2013	18	4.68
21-Nov-2012	22	53.16	21-Nov-2012	22	35.10
21-May-2013	22	25.31	20-Aug-2013	22	3.98
10-May-2010	24	39.79	15-Nov-2013	22	7.28
10-May-2010	24	52.97	21-May-2013	22	0.72
3-Aug-2011	28	54.89	3-Aug-2011	28	0.63
26-Jul-2012	29	3.46	26-Jul-2012	29	6.33
30-Oct-2012	40	86.15	26-Jul-2012	29	4.88
20-Sep-2012	70	265.02	28-Aug-2013	30	6.66
22-Jan-2010	79	432.75	20-Aug-2013	40	28.51
22-Jan-2010	79	392.89	30-Oct-2012	40	31.02
22-Jan-2010	79	458.86	24-Oct-2013	54	46.28
			24-Oct-2013	62	59.92
			20-Sep-2012	70	59.58
			20-Sep-2012	70	75.58

[1]Date represents when slides were removed
Greyed rows = DNA Extraction
