# Supplementary material for: Assembly and Succession of Iron Oxide Microbial Mat Communities in Acidic Geothermal Springs
Source: Front Microbiol. 2016 Feb 15;7:25. doi: 10.3389/fmicb.2016.00025 (PMC4753309; doi:10.3389/fmicb.2016.00025)
Supplement: Supplementary file 2 [file Presentation1.PDF]

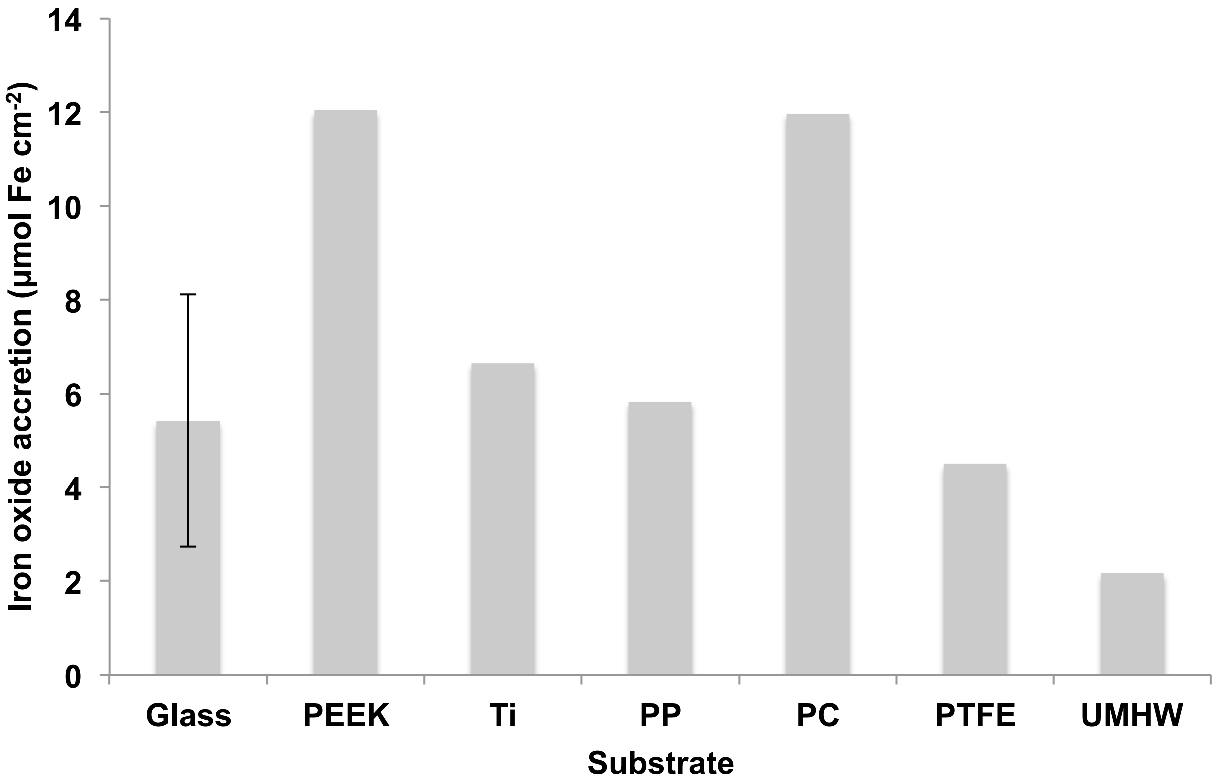

Figure S1. Effect of substrate on the accretion of iron oxides *in situ* at Beowulf Spring after 13 days of incubation (November 6, 2013). The range observed over different substrates was within the variation observed for borosilicate glass at time points near 10 - 14 d (glass, borosilicate glass; PEEK, polyether ether ketone; Ti, titanium; PP, polypropylene; PC, polycarbonate; PTFE, polytetrafluoroethylene; UMHW, ultra-high-molecular-weight polyethylene. Error bar on glass = 1 standard deviation (n=3).
